# A comparison of the efficacy and safety of complementary and alternative therapies for gastroesophageal reflux disease: A protocol for network meta-analysis

**DOI:** 10.1097/MD.0000000000021318

**Published:** 2020-07-24

**Authors:** Shixiong Zhang, Qian Jiang, Xiyan Mu, Zehou Wang, Shaowei Liu, Zeqi Yang, Miaochan Xu, Xuetong Ren, Yangang Wang

**Affiliations:** a Hebei University of Chinese Medicine; b Hebei Province Hospital of Chinese Medicine, Shijiazhuang City, Hebei, China; c Waters Corporation, MA; d Beijing University of Chinese Medicine, China.

**Keywords:** complementary and alternative therapies, gastroesophageal reflux disease, protocol, systematic review

## Abstract

**Background:**

Gastroesophageal reflux disease (GERD) is one of the most common gastrointestinal diseases in the world and is showing increasing prevalence in some countries. The disease has a chronic course that leads to a significant decline in the quality of life of patients and is associated with a high economic burden worldwide. And complementary and alternative medicine is used to treat the disease. Over the past few decades, a number of randomized controlled trials and systematic evaluations have been conducted to evaluate the effectiveness and safety of different types of complementary and alternative medicine methods, so there is an urgent need to summarize and further evaluate these studies.

**Methods:**

We will search the following sources without restrictions for date, language, or publication status: PubMed, Cochrane Central Register of Controlled Trials (CENTRAL) Cochrane Library, and EMBASE, China National Knowledge Infrastructure, Chinese Bio-medicine Database, VIP Chinese Periodical Database, Wan Fang Database. We will apply a combination of Medical Subject Heading and free-text terms incorporating database-specific controlled vocabularies and text words to implement search strategies. We will also search the ongoing trials registered in the World Health Organization's International Clinical Trials Registry Platform. Besides, the previous relevant reviews conducted on complementary and alternative therapies for GERD and reference lists of included studies will also be searched.

**Results:**

This study will provide a reliable basis for the treatment of GERD with complementary and alternative therapies.

**Conclusions:**

The findings will be an available reference to evaluate the efficacy and safety of complementary and alternative therapies on GERD and may provide decision-making reference on which method to choose for clinicians.

**PROSPERO registration number:**

PROSPERO CRD42020169332.

## 1 Introduction

According to the Montreal definition, gastroesophageal reflux disease (GERD) is defined as a disease which is associated with troublesome symptoms and/or complications on account of reflux of stomach contents into the esophagus. Most patients with GERD presents with heartburn and effortless regurgitation.^[1]^ It is one of the most common gastrointestinal diseases in the world, clinical diagnosis is made on the basis of typical symptoms, supported by symptom response from empiric proton pump inhibitor (PPI) therapy.^[2]^ It has been reported that the pooled prevalence of at least weekly GERD symptoms reported from population-based studies worldwide is approximately 13%, but there is considerable geographic variation.^[3,4]^ A systematic review demonstrated that the prevalence of GERD ranged from 18.1% to 27.8% in North America, 8.8% to 25.9% in Europe, 2.5% to 7.8% in East Asia, 8.7% to 33.1% in the Middle East, 11.6% in Australia, and 23.0% in South America.^[5]^ Evidence suggests an increase in GERD prevalence since 1995 (*P* < .0001), particularly in North America and East Asia.^[6]^ The disease has a chronic course that leads to a significant decline in the quality of life of patients and is associated with a high economic burden worldwide.^[7]^

Most patients with typical symptoms of GERD were treated with medication. Medical therapy includes: antacids, histamine 2 receptor antagonists, PPIs, Carafate, Transient Lower Esophageal Sphincter Relaxation reducer and prokinetics. Medication therapy for GERD is targeted at symptom reduction and minimizing mucosal damage from acid reflux. In particular, PPIs are considered to be the most effective drug for the treatment of GERD because of their profound and consistent acid inhibition. Although PPIs are currently the most effective treatment for GERD and its complications, up to 40% of patients with nonerosive reflux disease remain symptomatic on standard therapy, and approximately 10% to 15% of patients.^[8–10]^ Besides, it is reported that chronic PPI use is associated with an increase in the risk of gastric cancer. It might also be an independent risk factor for gastric cancer.^[11]^ The treatment option in this case is antireflux surgery. But it is not popular due to its invasive nature and potential adverse events.^[12]^ Therefore, more and more people are focusing on complementary and alternative therapies for the GERD.

Complementary and alternative therapies include exercise, acupuncture, moxibustion, Chinese herbal medicines, behavioral interventions, topical heat, dietary supplements, and so on. Many studies and reviews have proved that the patients with GERD would benefit by having complementary and alternative therapies for reducing pain treatment with minimal adverse effects. It has been reported that lifestyle changes play an important role in the treatment of GERD.^[13]^ One study showed that percutaneous electrical stimulation improved symptoms in patients with GERD by increasing Lower Esophageal Sph Incter Pressure and reducing weak acid reflux and acid reflux events.^[14]^ A randomized controlled trial (RCT) comparing the efficacy of wu chu yu tang and omeira for GERD found that wu chu yu tang for GERD was similar to omeprazole. Moreover, the effects of wu chu yu tang seem to last longer than those of prilosec.^[15]^

There are so many complementary and alternative therapies for GERD and their efficacy has been assessed by several systematic reviews. But the traditional pairwise meta-analyses only evaluated the direct comparison of pair-wised drugs and conflicting interpretation of results also existed among different studies. Therefore, the objective of this network meta-analysis (NMA) is to compare the complementary and alternative therapies in terms of the efficacy and safety for the treatment of GERD, and to better guide clinical practice and health policies.

## 2 Objective

This network meta-analysis aims to evaluate the current evidence for the efficacy and safety of complementary and alternative therapies for the GERD.

## 3 Methods and analysis

### 3.1 Study registering and reporting

The research will follow the preferred reporting items for systematic reviews and meta analyses (PRISMA) for NMA guidelines for reporting the results of the review. PROSPERO (international register of expectations system evaluation) (CRD42020169332) has registered this plan. We will record any protocol changes made during the implementation of the review in the publication of the final report. The PRISMA extension declaration is a declaration that ensures that all aspects of the method and result are reported. We followed the PRISMA-P guidelines.^[16,17]^

### 3.2 Eligibility criteria

The design of inclusion criteria and exclusion criteria in this study is based on the 5 main principles of PICOS.

#### 3.2.1 Type of participants

The inclusion of this study is adults with GERD (as diagnosed using any recognized diagnostic criteria). And the exclusion is adolescents (under 18 years of age) and elderly people (over 70).

#### 3.2.2 Type of interventions and comparators

Our intervention is complementary and alternative therapies, including exercise, acupuncture, moxibustion, herbal medicine, behavioral intervention, topical heat, dietary supplements, and so on. And, the control group included placebo, no treatment, and western medicine.

#### 3.2.3 Outcomes

The main outcomes included overall efficiency, reflux disease diagnostic questionnaire (RDQ) score, and symptom total score. And the additional outcomes included relapse rate and adverse reactions.

#### 3.2.4 Study design

In order to limit heterogeneity and enhance clinical applicability, strict inclusion/exclusion criteria were established. Only the RCTs associated with complementary and alternative therapies in GERD therapy were included for analysis. We will rule out repeated studies that do not have enough information to calculate effect estimates. We will not apply any language or other restrictions.

### 3.3 Information source

We will search the following sources without restrictions for date, language, or publication status: PubMed, Cochrane Central Register of Controlled Trials (CENTRAL) Cochrane Library, and EMBASE, China National Knowledge Infrastructure, Chinese Bio-medicine Database, VIP Chinese Periodical Database, Wan Fang Database. We will apply a combination of Medical Subject Heading (MeSH) and free-text terms incorporating database-specific controlled vocabularies and text words to implement search strategies. We will also search the ongoing trials registered in the World Health Organization's International Clinical Trials Registry Platform. Besides, the previous relevant reviews conducted on complementary and alternative therapies for GERD and reference lists of included studies will also be searched.

### 3.4 Search strategy

Two authors will screen the titles and abstracts of the all records retrieved in above electronic databases independently to find potentially eligible reviews. According to the inclusion and exclusion criteria outlined above, the full texts of them will be retrieved for further identification. Any disagreement will be resolved by discussion or by consultation with a third author. The search strategy for PubMed is presented in Table 1 and the strategy will be modified upon the requirement of other databases.

**Table 1:**
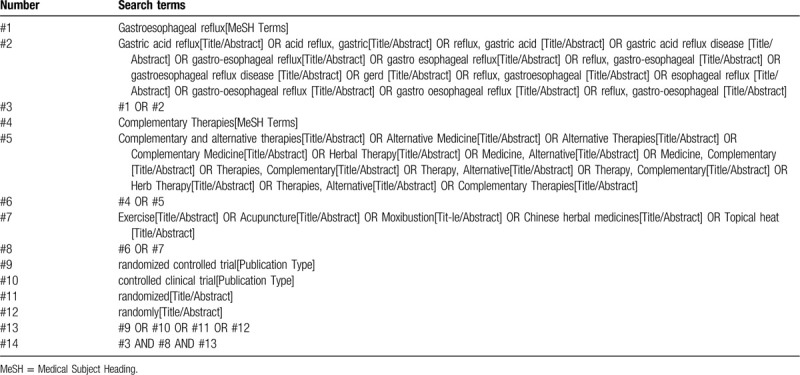
Search strategy used in PubMed database.

### 3.5 Data collection and analysis

#### 3.5.1 Study selection

Two reviewers will perform literature screening, study selection, and data extraction independently. The literature obtained will be imported into EndnoteX9 to screen the title and abstract, the duplications and studies failing to meet the prespecified inclusion criteria will be excluded. After reading the full text of the remained literature and discussing within the group, the final included studies will be determined. The corresponding author of original RCT will be contacted when the full text is unavailable. Disagreements will be solved by consulting a third-party arbitrator or discussing within a group.

#### 3.5.2 Data extraction and management

Two authors will screen the titles and abstracts of the all records retrieved in above electronic databases independently to find potentially eligible reviews. According to the inclusion and exclusion criteria outlined above, the full texts of them will be retrieved for further identification. Any disagreement will be resolved by discussion or by consultation with a third author.

Data will be extracted by 2 reviewers independently using a predesigned data extraction form. A third reviewer will validate data. The following data will be extracted: general information, trial characteristics, intervention(s) and control(s), participants, study methodology, outcomes, results, etc.

#### 3.5.3 Risk of bias in included studies

The methodological quality of eligible studies will be assessed by 2 review authors independently according to the Cochrane Handbook for Systematic Reviews of Interventions. The following characteristics will be assessed: random sequence generation (selection bias), allocation concealment (selection bias), blinding of participants and personnel (performance bias), blinding of outcome assessment (detection bias), incomplete outcome data (attrition bias), selective reporting (reporting bias), other bias. Based on the assessments of the studies against these 7 domains, they will be classified as being of “low risk,” “high risk” or “unclear risk” of bias. Any disagreements will be resolved by discussion or discussed with another reviewer if necessary.

#### 3.5.4 Data analysis

We will pool the results using a random-effects meta-analysis, with standardized mean differences for continuous outcomes, and calculate 95% confidence intervals and 2 sided *P* values for each outcome.

Heterogeneity between the studies in effect measures will be assessed using the *I*^2^ statistic, and we will consider an *I*^2^ value greater than 50% as being indicative of substantial heterogeneity.

We will conduct sensitivity analyses based on study quality.

We will use stratified meta-analyses to explore heterogeneity in effect estimates according to: study quality; study populations; the logistics of intervention provision; and intervention content. We will also assess evidence of publication bias.

We will perform a Bayesian NMA model for each outcome to estimate the overall treatment effects.

In our NMA, we will use WinBUGS 14.3 and Stata 14.0.

If results of the meta-analysis are significantly heterogeneous, subgroup analyses of the control groups might be performed.

#### 3.5.5 Patient and public involvement

This is a meta-analysis study based on previously published data, so patient and public involvement will not be included in this study.

#### 3.5.6 Grading the quality of evidence

The Grading of Recommendations Assessment, Development, and Evaluation (GRADE) guidelines will be utilized to grade the quality of evidence as very low, low, moderate, or high.

## 4 Discussion

GERD is one of the most common gastrointestinal diseases in the world and is showing increasing prevalence in some countries.^[18]^ The disease has a chronic course that leads to a significant decline in the quality of life of patients and is associated with a high economic burden worldwide.^[19]^ And complementary and alternative medicine is used to treat the disease.^[20]^ Over the past few decades, a number of RCTs and systematic evaluations have been conducted to evaluate the effectiveness and safety of different types of complementary and alternative medicine methods, so there is an urgent need to summarize and further evaluate these studies.^[21–23]^

This will be the first NMA to comprehensively compare the efficacy of complementary and alternative therapies for GERD. Despite the advantages of this approach, there are some inevitable limitations. Some therapies are not discussed in the literature due to the lack of RCTs or the RCT is still ongoing. The potentially high heterogeneity among different studies may also influence the final results of this NMA. However, we hope this study will uncover the best treatment currently available for clinical practice and assist in directing future study design.

## Author contributions

**Conceptualization:** Shixiong Zhang, Yangang Wang.

**Data curation:** Shixiong Zhang, Qian Jiang, Miaochan Xu.

**Formal analysis:** Zehou Wang, Xiyan Mu.

**Funding acquisition:** Yangang Wang.

**Methodology:** Shixiong Zhang, Shaowei Liu, Zeqi Yang, Miaochan Xu.

**Resources:** Yangang Wang

**Software:** Xuetong Ren, Zeqi Yang.

**Supervision:** Xiyan Mu.

**Writing – original draft:** Shixiong Zhang

**Writing – review & editing:** Miaochan Xu.
